# Comment on: *IL13* gene polymorphisms among Sudanese patients with bronchial asthma: a case-control study

**DOI:** 10.22099/mbrc.2025.52030.2082

**Published:** 2025

**Authors:** Zahra Zendehboodi

**Affiliations:** Department of Biology, School of Science, Shiraz University, Shiraz, Iran

**Dear Editor,**

I recently read an elegant study by Abdalla and Hamdan (2025) published in the *Molecular Biology Research Communications* (MBRC). The study aimed to investigate the association of two *IL13* polymorphisms including rs1800925 and rs20541 with bronchial asthma in Sudan [1]. The results showed that compared with TC genotype, TT genotype in rs1800925 significantly increased the risk of asthma (OR=3.15, 95% CI: 1.13-8.76; p=0.028), while no association was found with rs20541 [1]. 

However, there are some issues that need to be considered. First, Sudan has a heterogeneous population with different ethnic groups harboring inter-group genetic variation [2]. Therefore, the ethnicity of the participants should be determined. Second, since the two polymorphisms rs1800925 and rs20541 belong to the same gene (*IL13*), it is crucial to consider haplotype analysis when interpreting the data. 

As expected, moderate to tight linkage disequilibrium (LD) of these two single nucleotide polymorphisms has been reported in studies from other parts of the world [3, 4]. It was reported that rs1800925 was not associated with rhinoconjunctivitis, but the haplotype of its C allele with the A allele of rs20541 was significantly associated with the disease [3]. In the study of the association between severe respiratory syncytial virus infection and four polymorphisms in *IL4* (rs2243250) and *IL13* (rs1881457, rs1800925, and rs20541), the haplotype of four polymorphisms showed a much stronger association with the disease than that of each polymorphism individually [5]. Also, the association between bullous pemphigoid and the haplotype of rs2243250 in *IL4* with rs1800925 and rs20541 in *IL13* was stronger than that of rs2243250 alone [6]. These data support the need to perform haplotype analysis along with single polymorphism analysis in disease genetic studies. 

Moreover, another significance of haplotype analysis is to assess the LD patterns in different populations, which may be informative to identify the evolutionary history of humans [7]. For example, in a survey of haplotypes consisting of Alu deletion and short tandem repeat polymorphisms in the *CD4* gene in different populations, the results strongly support a common and recent African origin for all non-African communities [8]. Especially because of  few reports of the frequency of rs1800925 and rs20541 polymorphisms from Sudanese population, providing the data of haplotype analysis could be valuable for future disease association meta-analysis and evolutionary history studies.
